# Effects of task complexity or rate of motor imagery on motor learning in healthy young adults

**DOI:** 10.1002/brb3.2122

**Published:** 2021-10-06

**Authors:** Nargis Heena, Nayeem U. Zia, Stuti Sehgal, Shahnawaz Anwer, Ahmad Alghadir, Heng Li

**Affiliations:** ^1^ Max Smart Super Specialty Hospital New Delhi India; ^2^ Directorate of Health Services Kashmir Jammu and Kashmir India; ^3^ Institution of Rehabilitation Sciences, ISIC Vasant Kunj New Delhi India; ^4^ Rehabilitation Research Chair College of Applied Medical Sciences King Saud University Riyadh Saudi Arabia; ^5^ Department of Building and Real Estate Hong Kong Polytechnic University Kowloon Hong Kong Special Administrative Region

**Keywords:** motor imagery, motor learning, physical practice

## Abstract

**Background:**

A growing body of evidence suggests the benefit of motor imagery in motor learning. While some studies tried to look at the effect of isolated mental practice, others evaluated the combined effect of motor imagery and physical practice in clinical rehabilitation. This study aimed to investigate the effects of task complexity or rates of motor imagery on motor learning in health young adults.

**Methods:**

Eighty‐eight healthy individuals participated in this study. Participants were randomly allocated to either Group A (50% complex, *N* = 22), Group B (75% complex, *N* = 22), Group C (50% simple, *N* = 22), or Group D (75% simple, *N* = 22). Participants in the complex groups performed their task with nondominant hand and those in simple groups with a dominant hand. All participants performed a task that involved reach, grasp, and release tasks. The performance of the four groups was examined in the acquisition and retention phase. The main outcome measure was the movement time.

**Results:**

There were significant differences between immediate (i.e., acquisition) and late (i.e., retention) movement times at all three stages of task (i.e., MT_1_ [reaching time], MT_2_ [target transport time], and TMT [reaching time plus object transport time]) when individuals performed complex task with 75% imagery rate (*p* < .05). Similarly, there were significant differences between immediate and late movement times at all stages of task except the MT_2_ when individuals performed simple task with 75% imagery rate (*p* < .05). There were significant effects of task complexity (simple vs. complex tasks) on immediate movement time at the first stage of task (i.e., MT_1_) and late movement times of all three stages of task (*p* < .05). There were significant effects of the rate of imagery (50% vs. 75%) on late movement times at all three stages of tasks (*p* > .05). Additionally, there were no interaction effects of either task complexity or rate of imagery on both immediate and late movement times at all three stages of tasks (*p* > .05).

**Conclusion:**

This study supports the use of higher rates (75%) of motor imagery to improve motor learning. Additionally, the practice of a complex task demonstrated better motor learning in healthy young adults. Future longitudinal studies should validate these results in different patient's population such as stroke, spinal cord injury, and Parkinson's disease.

## Graphical abstract

This study aimed to investigate the effects of task complexity or rates of motor imagery on motor learning in health young adults. Participants were randomly allocated to either Group A (50% complex, *N* = 22), Group B (75% complex, *N* = 22), Group C (50% simple, *N* = 22), or Group D (75% simple, *N* = 22). The performance of the four groups was examined in the acquisition and retention phase. There were significant differences between immediate (i.e., acquisition) and late (i.e., retention) movement times at all three stages of task when individuals performed complex task with 75% imagery rate (*p* < .05). Similarly, there were significant differences between immediate and late movement times at all stages of task except the transport time when individuals performed simple task with 75% imagery rate (*p *< .05). This study supports the use of higher rates (75%) of motor imagery to improve motor learning. Additionally, the practice of a complex task demonstrated better motor learning in healthy young adults.

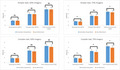

## 1 BACKGROUND

Motor imagery, also known as “mental practice,” is an imagery training of a motor task to improve learning without giving any simultaneous sensory input or apparent output (Jackson et al., 2001). This training involves kinesthetic and visual information about a specific movement that is mentally practiced (Decenty, 1993). Imagery refers to the creation (or recreation) of any experience in mind—auditory, visual, kinesthetic, or organic. Motor imagery is the mental portrayal of a specific movement in the absence of any apparent movement (Ji et al., 2014). It is an autonomous composite cognitive process that uses sensory and perceptual processes to reactivate any specific actions inside the operational memory (Ji et al., 2014). Mental practice is the self‐practice of imagery tasks, while motor imagery practice is referred to the mental practice of motor imagery contents aimed to enhance motor performance. Motor imagery practice and mental practice often are used interchangeably (Dickstein & Deustch, 2007).

A large body of evidence suggests that motor imagery may enhance the learning of motor tasks (Decenty, 1993). While many studies have reported the effectiveness of mental practice to optimize movement execution in athletes (Jeannerod, 1994; Malouin & Richards, 2010; Mulder et al., 2004; Warner & McNeill, 1988), others suggested a positive influence of motor imagery on neural mechanisms (Kosslyn et al., 2001; Pearson et al., 2015; Sirigu & Duhamel, 2001). According to Paivio (2014), the motivational factors that cause physiological arousal might play a vital role to improve motor performance after motor imagery. Additionally (Sackett, 1934) suggests a symbolic learning theory, which reveals that mental practices can encourage motor task by permitting subjects to practice the cognitive segments of a task. According to this theory, the movements are coded symbolically in the central nervous system, consequently easier to execute them when required. A previous study reported a functional redistribution after motor imagery training in healthy adults suggested that motor imagery training alone appears to promote the modulation of neural circuits, causing plastic changes in the motor system similar to the changes seen after repeated physical practice (Ietswaart et al., 2006). Another study reported that motor imagery produces similar electroencephalographic patterns and may activate a brain network likewise shown by the execution of real tasks (Caldara et al., 2004).

The use of motor imagery and mental practice by physical therapists may have many positive implications. For instance, Richardson (Richardson, 1967) suggested that a specific mental practice may be useful in a selected patient, such as perceptual motor disability. Since motor imagery requires no special equipment and easy to teach and learn, it may readily be adapted by physical therapists in their clinical practice (Warner & McNeill, 1988). Although evidence for the effectiveness of motor imagery to enhance new motor skills is inconclusive, there is general agreement that motor imagery may help improve performance, particularly in combination with real physical practice (Yágüez et al., 1998).

A growing body of evidence suggests the benefit of motor imagery in motor learning (Ruffino et al., 2017). While some studies tried to look at the effect of isolated mental practice, others evaluated the combined effect of motor imagery and physical practice in clinical rehabilitation. For example, while Page et al. (2005) and Jackson et al. (2004) have used a low rate of motor imagery and more physical practice, the Gaggioli et al. (2004) used an equal amount of motor imagery and physical practice. Pascual‐Leone et al. (1995) have compared motor imagery and physical practice of piano exercise, and they concluded that the level of performance after 5 days of motor imagery training was equivalent to that of 3 days of physical practice. The addition of one physical practice session to the 5 days of motor imagery training resulted in a similar level of performance in motor imagery and physical practice groups. However, Allami et al. (2008) showed that motor imagery practice at high rates results in significantly better performance compared to physical practice alone, particularly if the task was complex or difficult. Notably, when the mental practice of 50% or 75% of the trials was rehearsed, the performance of the first executed trial was significantly better than that of those in the physical practice group, suggesting that mental practice is better than no practice at all (Allami et al., 2008). However, the results of this study were limited due to the small sample size and there was difference in the performance of movement time between the two groups at baseline (Allami et al., 2008).

The prerequisite for motor learning through mental or physical practice requires the task should be unknown or more complex than the individual's capacity (Allami et al., 2008). The “complex task” is often used interchangeably with “difficult task” in the previous literature of motor learning (Verstynen et al., 2005; Wulf et al., 1998). For instance, a task can be categorized as complex or difficult if the movement or reaction times are comparatively long, if a training program requires long practice hours, or if the task requires a very high demands of attention and memory of the learners (Wulf & Shea, 2002). In the current study, the task was labeled as “complex” when it was performed by nondominant hand and the task was labeled as “simple” when it was performed by the dominant hand. The performance of a task by nondominant hand may pose high demands on the attention of individuals in several ways (Klaming & Vlaskamp, 2018). For example, a previous study indicates that the performance of a motor task is often slower with the nondominant hand, which is mainly due to reduced movement accuracy and increased need of corrective movements (Annett et al., 1979). Additionally, many studies indicate an increased interference effect due to increased cognitive loading during a dual task (e.g., simultaneous performance of cognitive and motor tasks) performance when the motor task was performed by a nondominant hand (Strenge & Niederberger, 2008; Theill et al., 2011; Yamashita, 2010).

Therefore, the primary objective of this study is to investigate the difference between different rates of motor imagery on motor learning in normal young adults. Secondarily, it aims to investigate the relationship between complexity of the task with the motor imagery rates.

## 2 METHODS

### 2.1 Participants

**FIGURE 1:**
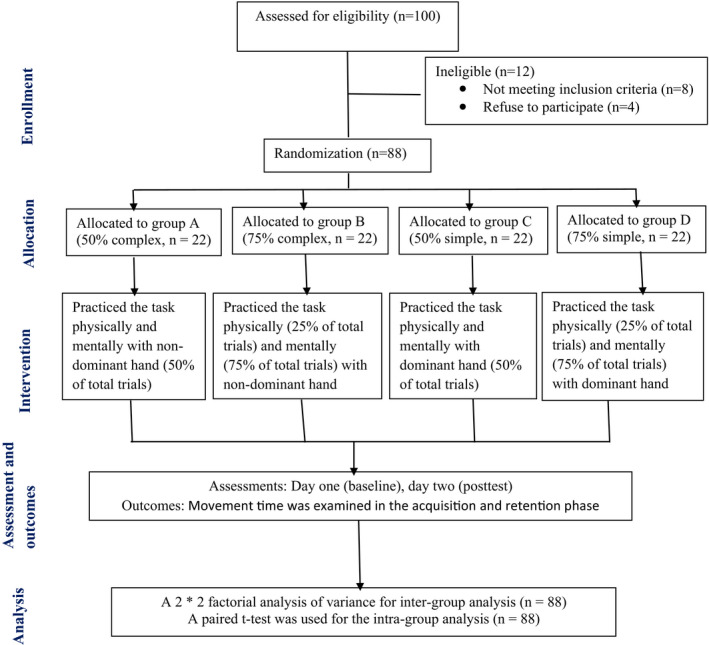
Flow diagram for study procedure of the randomized trial

A total number of 88 individuals were selected for the study, and 22 subjects were allocated to each group, namely Group A (50% complex task), Group B (75% complex task), Group C (50% simple task), and Group D (75% simple task). These individuals were the ones who were eligible and consented to participate apart from 12 other individuals who were dropouts. The subjects were randomly allocated to each group using a lottery method (Figure 1). The inclusion criteria were as follows: (a) Healthy young adults; (b) Both Genders; (c) Age group: 20–30 years; (d) Movement imagery Questionnaire‐Revised second version (Hall & Martin, 1997) score ≥5 because lesser scores limit one's ability to imagine. A pre‐post experimental design was used in the study. A closed environment with the least possible distraction was maintained during the data collection. All participants were instructed about the procedures used in the study. The participants received detailed information, and an informed consent approved by the ethics committee, Jamia Hamdard, New Delhi, was taken.

### 2.2 Procedures

Forty individuals were selected for the preliminary experiment, twenty in each of the two groups, that is, simple and complex task groups. All participants performed a task overtly that involved reach, grasp, and release. The purpose of this preliminary experiment was to determine the number of trials required to learn a specific task. Through this experiment, a classical learning curve with a progressive decrease in movement time, suggestive of learning and finally a plateau corresponding to the stabilization of the best performance, was obtained. The average number of trials calculated from the simple and complex group was 90 and 108, respectively. Individuals who participated in the pilot experiment did not participate in the actual study. The pilot data generated were used only to determine the average number of trials required to learn the task.

After determining the average number of trials, a total number of 88 individuals were selected for the study and 22 subjects were allocated to each group, namely Group A (50% complex task), Group B (75% complex task), Group C (50% simple task), and Group D (75% simple task). Participants in the complex groups performed the task with their nondominant hand and those in simple groups with their dominant hand.

### 2.3 Task and performance

**FIGURE 2:**
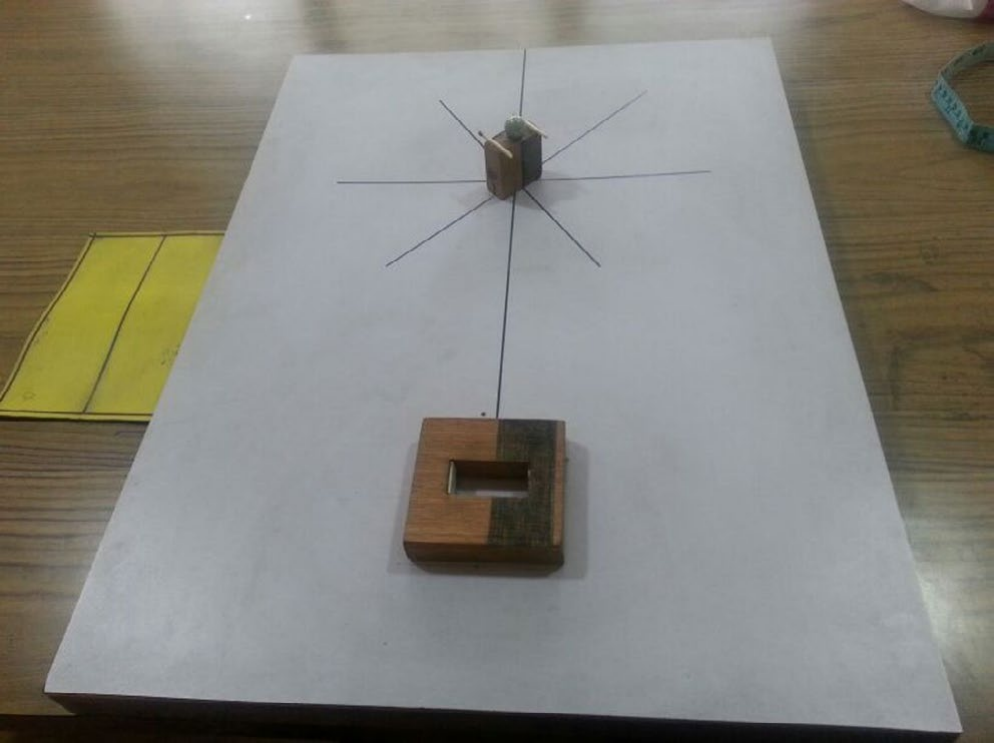
The task set‐up

The motor task used in the actual experiment was the same as used for the pilot experiment. The participants in each group were comfortably seated and fastened to the adjustable chair facing toward the table, with the dominant hand in the case of simple and nondominant in the case of complex, resting the palm at the starting position marked on the table, located 29 cm (reference point) from the sagittal axis with the tip of the middle finger touching the edge of the sheet (Figure 2). Height of the chair was adjusted so that the tabletop corresponded to the xiphisternum of each participant. Each participant was instructed to reach and grasp a wooden rectangular target with a marble on top of it to transport it and finally insert it into a support as fast as possible. The marble was placed on a slight but shallow hole made on the surface of the target. Furthermore, two small wooden sticks were glued to the smaller sides of the target to force the participants to grasp it with a precision grip. The target was located along the participants’ sagittal axis at 50 cm from the chest. Half of the target's surface was colored in black, while the other half was left wooden, which were matched with identical marks on the support by the participants while placing the target in the support. The target was kept at an angle of 45 degrees with a reference line (perpendicular to the support) to increase difficulty in task execution. If the marble fell from the top of the target at any point of the task, the trial was discarded.

**FIGURE 3:**
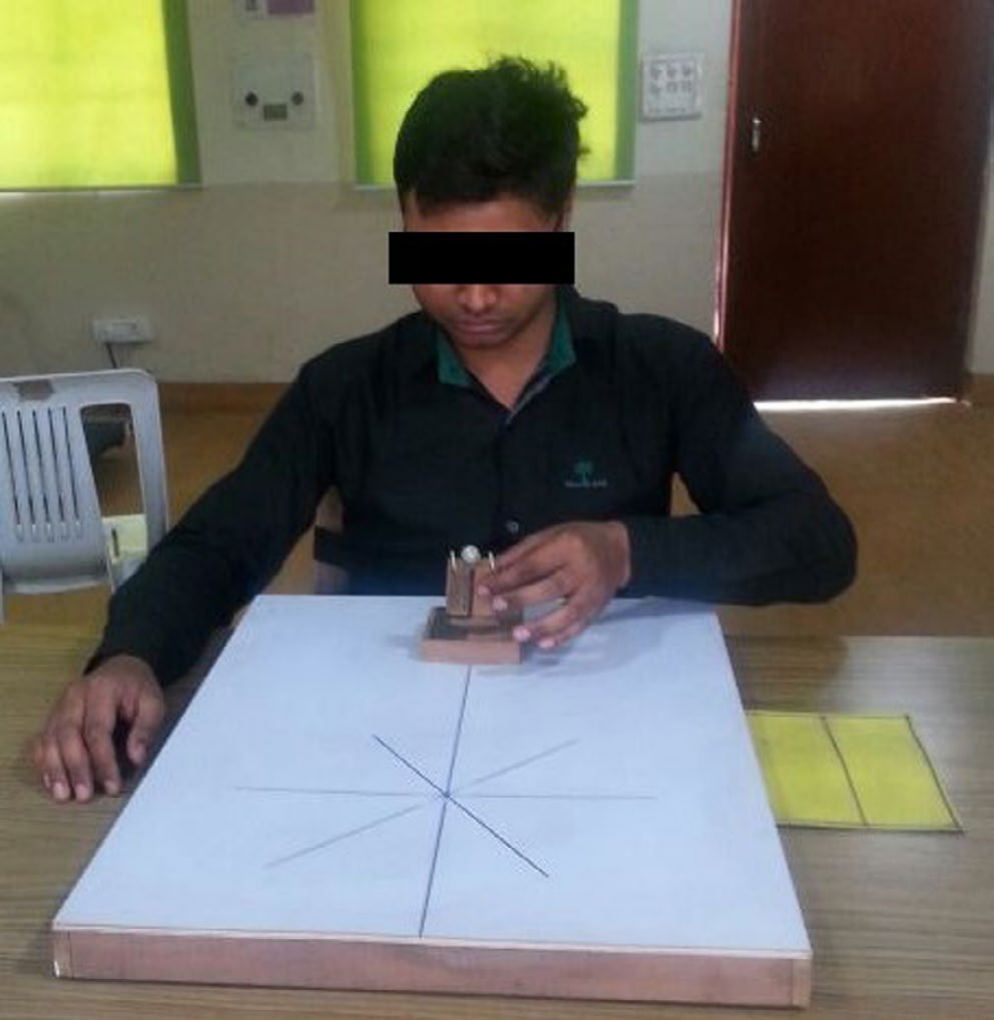
A participant performing task physically

**FIGURE 4:**
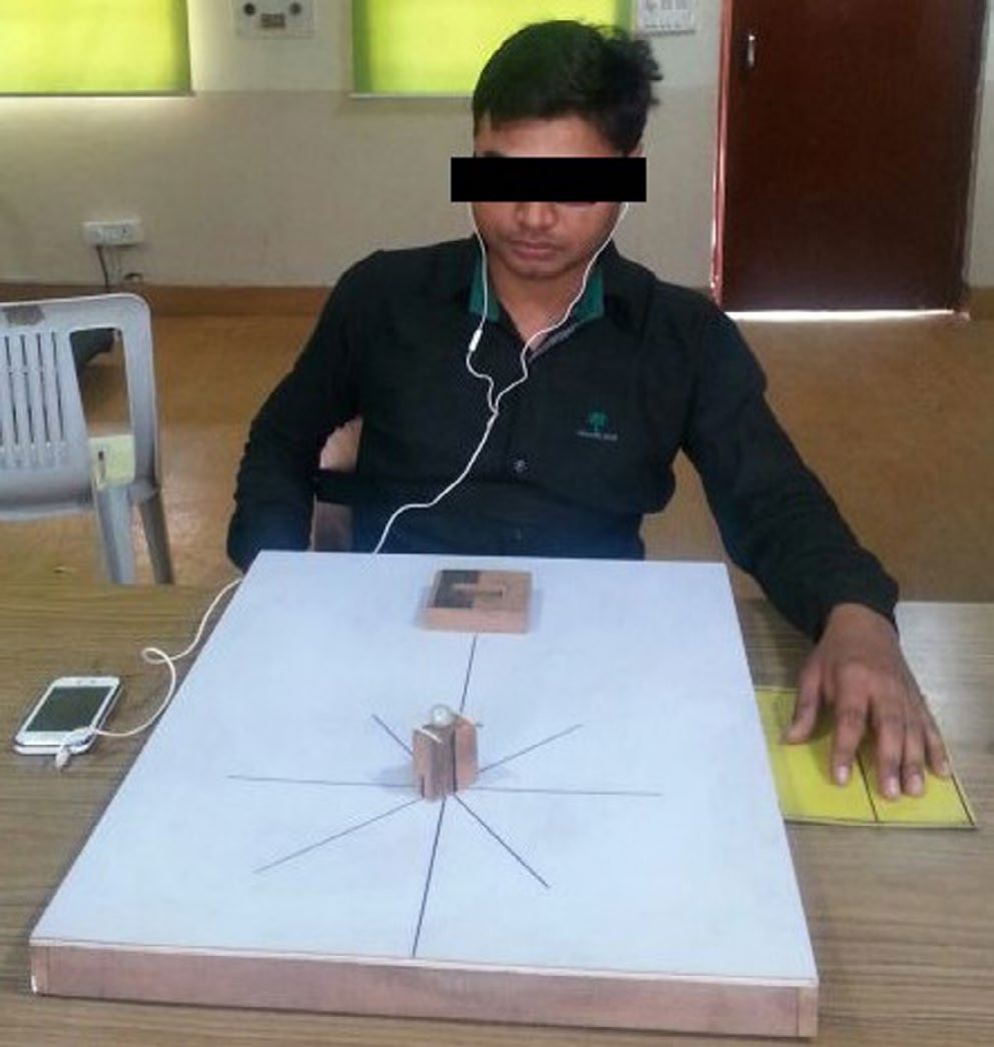
A participant performing task mentally

Participants in all groups were made to rehearse the task physically (Figure 3) and mentally (Figure 4) at different rates. The type of command used in motor imagery was auditory, which was administered using an audio clip. The audio clip consisted of commands to make each participant to rehearse the task mentally. Participants in Group A practiced the task physically and mentally with the nondominant hand at the same rate of 50% of the total number of trials, that is, they rehearsed the task 54 times mentally and 54 times physically. Participants in Group B practiced the task physically and mentally with their nondominant hand at different rates of 25% and 75%, respectively, that is, they rehearsed the task 81 times mentally and 27 times physically. The participants in Group C practiced the task physically and mentally with their dominant hand at the same rate of 50% of the total number of trials, that is, they rehearsed the task 45 times mentally and 45 times physically. The participants in Group D practiced the task physically and mentally with the dominant hand at different rates of 25% and 75%, respectively, of the total number of trials, that is, they rehearsed the task 67 times mentally and 23 times physically. The participants practiced the task in a blocked fashion. The order was constant across the individuals.

### 2.4 Measurements

**FIGURE 5:**
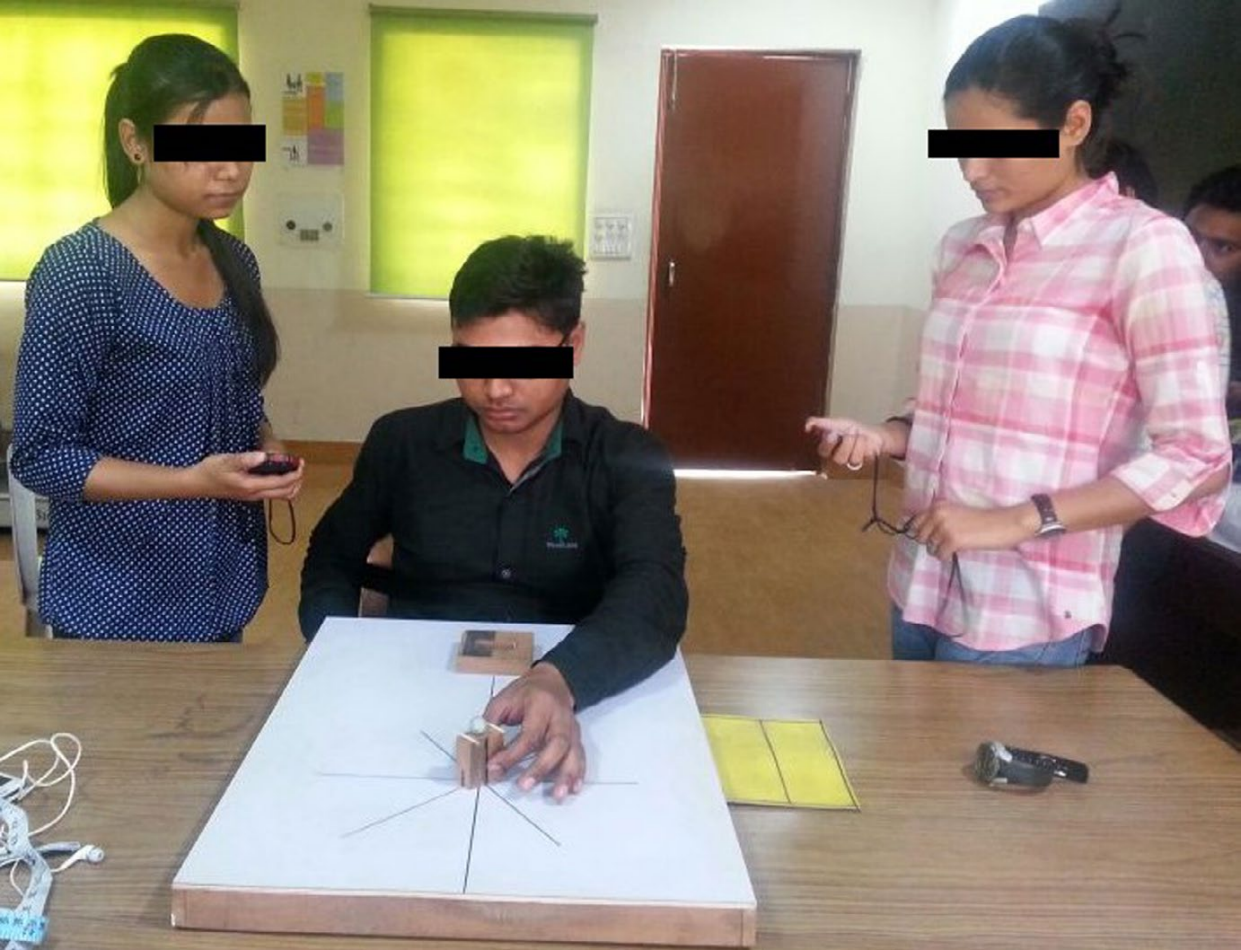
Measurements being taken using a handheld stopwatch

On the first day, participants in each group were made to perform the task at their respective rates of physical and mental performance. Enough rest periods were given after physical practice and mental practice. Blood pressure and heart rate of the participants were measured before and after the mental practice session, to ensure their engagement in the mental rehearsal (Decenty, 1993). To check the acquisition, an average of three measurements of total movement time (TMT), movement time one (MT1), and movement time two (MT2) were taken in seconds. Total movement time was measured as the sum of movement time one (MT1) and movement time two (MT2). MT1 was the time taken by the participants to reach and grasp the target and MT2 the time taken to pick up the target and insert it into the support. Two aspects of movement time would provide an insight into as to how different rates of motor imagery and physical practice influence the complexity of the task. MT1 is an indicator for the transport of arm‐hand complex, which is simpler than the grasp and manipulation, indicated by MT2. Time measurements were taken using a handheld stopwatch which is a reliable method (Figure 5; Hetzler et al., 2008).

To check the retention and learning, the same measurements were repeated on the second day. Precisely, the practice and retention sessions were separated by one night of sleep because researches suggest that sleep leads to better consolidation of practiced events which is critical for learning (Song, 2009). A comparison between the measurements of two different days was used for the analysis. Measurements were taken by the same therapists who were blinded to the allocation process completely.

### 2.5 Statistical analysis

Data were analyzed using SPSS for Window (version 22, IBM). Normality of the data was verified using the Shapiro–Wilk test (*p* >.05). Intragroup analysis was done using the paired *t* test. Intergroup comparison was done using the 2 × 2 factorial analysis of variance (ANOVA) test using imagery rate (50% vs. 75%) and complexity of tasks (simple vs. complex tasks) as factors. A *p* <.05 was considered statistically significant. Sample size was calculated using the G*Power 3.1.9.4 power analysis software. The movement time score with the power of 80% (*F* test) and the alpha 0.05 (two‐tailed) were used for calculating the sample size. The required sample was 21 in each group (total sample = 84) with effect size of 0.20.

### 2.6 Ethical approval

This study was approved by the ethics committee, Jamia Hamdard, New Delhi. Consent to participate: An informed consent has been obtained from all participants.

## 3 RESULTS

### 3.1 Demographics

Eighty‐eight subjects recruited for the study were randomly allocated into four groups. Group A consisted of 22 participants (12 females and 10 males) with mean age 23.18 ± 1.94 years, Group B consisted of 22 participants (11 females and 11 males) with mean age 23.41 ± 2.52 years, Group C consisted of 22 participants (12 females and 10 males) with mean age 23.55 ± 2.63 years, and Group D consisted of 22 (12 females and 10 males) with mean age 23.0 9 ± 2.97 years. There was no significant difference in age (*F* = 0.14, *p* =.93) among all groups.

### 3.2 Intragroup comparison of movement time in each group

Intragroup comparison of movement time in the four groups is given in Table 1. There were no significant differences between immediate (i.e., acquisition) and late (i.e., retention) movement times at all three stages of task (i.e., MT_1_ [reaching time], MT_2_ [target transport time], and TMT [reaching time plus object transport time]) when individuals performed task with 50% imagery rate irrespective of task complexity (i.e., simple or complex). There were significant differences between immediate (i.e., acquisition) and late (i.e., retention) movement times at all three stages of task (i.e., MT_1_ [reaching time], MT_2_ [target transport time], and TMT [reaching time plus object transport time]) when individuals performed complex tasks with 75% imagery rate. Similarly, there were significant differences between immediate (i.e., acquisition) and late (i.e., retention) movement times at all stages of task except the MT_2_ (target transport time) when individuals performed simple task with 75% imagery rate.

**TABLE 1:** Intragroup comparison of immediate (acquisition time) and late (retention time) movement times

Groups	Movement time		Mean	*SD*	*df*	*t*‐value	*p*‐value
Complexity: complex Imagery: 50% imagery (*n* = 22)	MT1	Immediate	0.91	0.17	21	0.567	.577
		Late	0.89	0.16			
	MT2	Immediate	2.38	0.38	21	1.642	.116
		Late	2.22	0.34			
	TMT	Immediate	3.29	0.46	21	1.645	.115
		Late	3.10	0.39			
Complexity: complex Imagery: 75% imagery (*n* = 22)	MT1	Immediate	0.94	0.18	21	3.269	.004*
		Late	0.85	0.14			
	MT2	Immediate	2.30	0.39	21	2.354	.028*
		Late	2.10	0.40			
	TMT	Immediate	3.24	0.43	21	3.326	.003*
		Late	2.95	0.43			
Complexity: simple Imagery: 50% imagery (*n* = 22)	MT1	Immediate	0.97	0.15	21	0.128	.899
		Late	0.97	0.16			
	MT2	Immediate	2.35	0.41	21	0.283	.780
		Late	2.32	0.35			
	TMT	Immediate	3.31	0.48	21	0.214	.832
		Late	3.29	0.44			
Complexity: simple Imagery: 75% imagery (*n* = 22)	MT1	Immediate	1.06	0.16	21	3.337	.003*
		Late	0.96	0.15			
	MT2	Immediate	2.47	0.56	21	0.922	.367
		Late	2.40	0.48			
	TMT	Immediate	3.53	0.67	21	2.042	.049*
		Late	3.36	0.55			

Abbreviations: MT_1_, reaching time; MT_2_, target transport time; TMT, reaching time plus object transport time.

* Significant at *p* <.05.

### 3.3 Intergroup comparison of movement time among the groups

Intergroup comparisons of movement time were compared using imagery rate (50% vs. 75%) and complexity of the task (simple vs. complex tasks) as factors, as shown in Table 2. There were significant effects of task complexity (simple vs. complex tasks) on immediate (i.e., acquisition) movement time at the first stage of task (i.e., MT_1_, reaching time) and the late (i.e., retention) movement times at all three stages of task (i.e., MT_1_ [reaching time], MT_2_ [target transport time], and TMT [reaching time plus object transport time]). There were significant effects of the rate of imagery (50% vs. 75%) on the late (i.e., retention) movement times at all three stages of task. Additionally, there were no interaction effects of either task complexity (simple vs. complex tasks) or rate of imagery (50% vs. 75%) on both immediate (i.e., acquisition) and late (i.e., retention) movement times at all three stages of task.

**TABLE 2:** The 2 × 2 factorial analysis of variance (ANOVA) test using imagery rate (50% vs. 75%) and complexity of tasks (simple vs. complex tasks) as factors

Variables	Source	*df*	Partial *ŋ* ^2^	*F*‐value	*p*‐value
MT_1_ ^a^	Complexity (simple vs. complex tasks)	1	0.069	6.226	.015*
	Rate of imagery (50% vs. 75%)	1	0.037	3.229	.076
	Complexity × rate of imagery	1	0.010	0.866	.355
MT_2_ ^a^	Complexity (simple vs. complex tasks)	1	0.006	0.504	.480
	Rate of imagery (50% vs. 75%)	1	0.001	0.054	.817
	Complexity × rate of imagery	1	0.013	1.106	.296
TMT1^a^	Complexity (simple vs. complex tasks)	1	0.022	1.923	.169
	Rate of imagery (50% vs. 75%)	1	0.007	0.592	.444
	Complexity × rate of imagery	1	0.016	1.385	.243
LMT_1_ ^b^	Complexity (simple vs. complex tasks)	1	0.096	8.911	.004*
	Rate of imagery (50% vs. 75%)	1	0.005	4.440	.037*
	Complexity × rate of imagery	1	0.002	0.185	.668
LMT_2_ ^b^	Complexity (simple vs. complex tasks)	1	0.064	5.726	.019*
	Rate of imagery (50% vs. 75%)	1	0.001	4.044	.034*
	Complexity × rate of imagery	1	0.015	1.287	.260
LTMT^b^	Complexity (simple vs. complex tasks)	1	0.102	9.545	.003*
	Rate of imagery (50% vs. 75%)	1	0.002	4.176	.036*
	Complexity × rate of imagery	1	0.015	1.305	.257

Abbreviations: MT_1_, reaching time; MT_2_, target transport time; TMT, reaching time plus object transport time.

^a^ Immediate (acquisition time).

^b^ Late (retention time).

* Significant at *p* <.05.

## 4 DISCUSSION

This study aimed to evaluate the effect of different rates of motor imagery and physical practice in motor learning. The results of this study indicate that a higher rate (e.g., 75%) of motor imagery practice demonstrated better motor learning compared to those who received lower rates (e.g., 50%) of motor imagery training. Additionally, individuals who practiced complex tasks improved motor learning much better than those who practiced simple tasks.

Likewise, a previous study reported a significantly better performance in the group who practiced higher rates of motor imagery (e.g., 50% or 75%) in combination with physical practice compared to physical practice alone, particularly when the task was more complex (Allami et al., 2008). Additionally, a recent study indicates that motor imagery and movement execution training were equally effective for enhancing sensorimotor performance during the acquisition of motor skills in healthy young adults (Bonassi et al., 2020). Other studies also suggested that a single session of motor imagery practice promotes motor learning, especially for the most complex task (Avanzino et al., 2009; Debarnot et al., 2010).

The current study found that the practice of complex tasks in motor imagery gives better motor learning. Similarly, a previous study also suggested that motor imagery of a complex task resulted in better motor learning in healthy adults (Allami et al., 2008). More recently, (Mashat et al., 2019) examined the effects of task complexity on motor imagery function using the brain–computer interface. They concluded that the classification accuracy of the brain–computer interfaces for a complex motor imagery task was 7.3% higher than the simple task. Researchers believed that a complex task needs a specific control of the limb muscles and proper motor planning, which require the participation of a large number of peripheral nerves (Mashat et al., 2019). Consequently, this might affect the location and the intensity of activation in the brain (Alahmadi et al., 2016). Since more complex tasks may possibly engages a larger area of brain due to its higher processing demands than the relatively simple task (Mashat et al., 2019), motor imagery of complex tasks produces better motor learning.

Motor imagery, which involves no overt movements, has been proven to activate the same areas of the brain as that of the actual movements (Kilteni et al., 2018). A growing body of evidence recommends motor imagery practice to improve motor performance and learning both in healthy individuals and people with health‐related issues including stroke, spinal cord injury, and Parkinson's disease (Dobkin, 2004; Hardy & Callow, 1999; Liu et al., 2004; Nyberg et al., 2006; Page et al., 2001). Additionally, past studies also indicate that a combination of motor imagery practice and physical practice or motor imagery practice alone may enhance motor learning both in healthy individuals and people with health‐related issues (Guillot & Collet, 2005; Michelon et al., 2006; Rushall & Lippman, 1997; Sharma et al., 2006). For example, motor imagery practice demonstrated increased performance for various motor control tasks, such as increased force production capacity of the selected muscles (Sidaway & Trzaska, 2005; Zijdewind et al., 2003), improved speed of arm movement (Gentili et al., 2006), increased range of motion (Williams et al., 2004), and improved postural control (Fansler et al., 1985; Hamel & Lajoie, 2005) in healthy individuals. Similarly, motor imagery practice has been successfully used to enhance performance accuracy (Blair et al., 1993; Yue & Cole, 1992), speed (Blair et al., 1993; Boschker et al., 2000), movement dynamics, muscle strength (Blair et al., 1993; Yaguez et al., 1998), and motor skills performance in athletes (Taktek, 2004). Likewise, numerous studies examined the effects of motor imagery practice in combination with physical therapy or motor imagery alone in people with various neurological ailments (Cramer et al., 2006; Malouin et al., 2004; Page et al., 2001; Tamir et al., 2007). For instance, a few studies demonstrated significant improvements in motor performance after a combined intervention of motor imagery practice and physical therapy compared with physical therapy alone in people with stroke (Malouin et al., 2004; Page et al., 2001). Additionally, a past randomized controlled study indicates significant improvements in daily function after combined motor imagery and physical practice training compared to physical therapy alone in people with Parkinson's disease (Tamir et al., 2007). Therefore, motor imagery practice may be an excellent alternative for improvement of function, particularly in individuals who have limited ability.

The current study has some potential limitations. The findings revealed in this study cannot be held valid for the patient population unless tested. Future research is warranted to further validate this result in patients with neurological pathologies (e.g., stroke) to know whether higher rates of motor imagery are better than lower rates of motor imagery in motor relearning. Additionally, movement times in the current study were not measured automatically, but manually by the examiners. Transfer testing could have been done in addition to the acquisition and retention testing. Moreover, kinematic analysis can be done to document the quality of the movement performance.

## 5 CONCLUSIONS

Motor imagery is an effective tool that can be used as an adjunct to physical training, and higher rates can also be used to partly replace physical practice, particularly when the task is complex. Additionally, higher rates of motor imagery possibly provide the opportunity to patients to reduce the level of physical activity and may reduce the chances of fatigue development. Future longitudinal studies are needed to further validate these results in different patient's population such as stroke, spinal cord injury, and Parkinson's disease.

## CONFLICT OF INTEREST

None declared.

## AUTHOR CONTRIBUTION

NH, NZ, SS, SA, AA, and HL involved in conceptualization and methodology. NH, NZ, and SS involved in data acquisition, investigation, original draft preparation, visualization, and validation. SA involved in data analysis and interpretation. SA, AA, and HL involved in reviewing, revising, and editing the manuscript. AA involved in funding acquisition and supervision.

### PEER REVIEW

The peer review history for this article is available at https://publons.com/publon/10.1002/brb3.2122.

## Data Availability

The data related to the findings of this study are available from the first author upon reasonable request.
